# Fluoropolymer-Containing Opals and Inverse Opals by Melt-Shear Organization

**DOI:** 10.3390/molecules24020333

**Published:** 2019-01-17

**Authors:** Julia Kredel, Christian Dietz, Markus Gallei

**Affiliations:** 1 Ernst-Berl Institute of Technical and Macromolecular Chemistry, Technische Universität Darmstadt, Alarich-Weiss-Straße 4, 64287 Darmstadt, Germany; j.kredel@mc.tu-darmstadt.de; 2 Institute of Materials Science, Physics of Surfaces, Technische Universität Darmstadt, Alarich-Weiss-Str. 2, D-64287 Darmstadt, Germany; dietz@pos.tu-darmstadt.de

**Keywords:** fluoropolymers, melt-shear organization, chemical resistance, solvent responsiveness, hydrophobicity, core/shell particles, emulsion polymerization, particle processing

## Abstract

The preparation of highly ordered colloidal architectures has attracted significant attention and is a rapidly growing field for various applications, e.g., sensors, absorbers, and membranes. A promising technique for the preparation of elastomeric inverse opal films relies on tailored core/shell particle architectures and application of the so-called melt-shear organization technique. Within the present work, a convenient route for the preparation of core/shell particles featuring highly fluorinated shell materials as building blocks is described. As particle core materials, both organic or inorganic (SiO_2_) particles can be used as a template, followed by a semi-continuous stepwise emulsion polymerization for the synthesis of the soft fluoropolymer shell material. The use of functional monomers as shell-material offers the possibility to create opal and inverse opal films with striking optical properties according to Bragg’s law of diffraction. Due to the presence of fluorinated moieties, the chemical resistance of the final opals and inverse opals is increased. The herein developed fluorine-containing particle-based films feature a low surface energy for the matrix material leading to good hydrophobic properties. Moreover, the low refractive index of the fluoropolymer shell compared to the core (or voids) led to excellent optical properties based on structural colors. The herein described fluoropolymer opals and inverse opals are expected to pave the way toward novel functional materials for application in fields of coatings and optical sensors.

## 1. Introduction

Fluor-containing polymers represent a unique class of functional materials combining different interesting properties, and such polymers have attracted significant attention in the recent past. Some of these properties are their remarkable resistance toward chemicals, high thermal stability, wetting behavior, and repellent capabilities, as well as their low refractive indices compared to other polymer materials [1,2,3]. Therefore, the field of applications for fluoropolymers is widespread and range over coatings, membranes, optical applications, and high performance elastomers [4,5,6,7,8]. Designing novel fluoropolymer and hierarchical architectures is relevant for the development of improved and new applications, for instance, in the field of three-dimensionally ordered porous coatings and photonic materials. In general, the control and understanding of surface properties is crucial for the development of advanced materials with well-defined wetting properties. Such so-called *smart surfaces* have already been used in applications such as self-cleaning surfaces, tunable optical lenses, lab-on-chip systems, microfluidic devices, and many different textile applications [9,10,11,12,13,14]. Considerable effort has been carried out on understanding the influence of designed, rough surfaces on the wetting properties, initiated by the pioneering works of Wenzel, Cassie, and Baxter long ago [15,16]. Hierarchical particle-based architectures, such as colloidal crystals and inverse opals with adjustable dimensions, have gained considerable attention due to their tremendous potential for various applications in catalysis, separation, sensors, optics, and biomedicine [17,18,19,20,21,22,23,24,25,26,27]. In the case of porous materials, different templating approaches have been used for the design of the final materials after removal of the template material [28,29,30,31,32,33]. Colloidal crystals can be prepared by various techniques such as particle deposition or spin coating of respective particle dispersions [21,34]. For example, Kim et al. developed omniphobic inverse opals for the preparation of omniphobic porous materials some years ago [35]. Vogel et al. reported on the infiltration of inverse opals with lubricants for gaining access to highly repellent surfaces toward many different liquids [36]. By this elegant approach, the adsorption of liquid-borne contaminants could be prevented, and reduction of ice adhesion could be accomplished. Single and dual inverse opal structures of fluoropolymer-containing materials have been developed by Wu and co-workers [37]. All these materials were obtained by the vertical deposition method or via spin-coating of particles. Another technique for the precise arrangement of polymer-based particles can be accomplished in flow fields by, e.g., combinations of melting and shear-ordering methods leading to so-called polymer opal films [27,38,39]. This so-called *melt-shear organization* technique requires core-shell particles and features the major advantage of fully solvent- and dispersion-free material processing. The hard core/soft shell particles are compressed between the plates of a moderately hot press, and the hard core particles can merge into the colloidal crystal structure yielding free-standing polymer opal films in one single step. Only recently, the feasibility of this technique was shown for inorganic core particles featuring a polymer or hybrid soft and meltable shell was reported [40,41,42,43,44]. This melt-shear organization technique allows for the facile preparation of almost perfectly ordered core/shell particles embedded in an elastomeric polymer matrix, and it can be applied on industrially relevant length scales [45]. The combination of fluoropolymers with this technique has not been reported for the preparation of functional opal films or inverse opals films yet. Within the present study, we report for the first time the incorporation of fluoropolymers into core/shell particle architectures, which can be advantageously used for the melt-shear organization technique. Both organic particle cores and inorganic silica core particles featuring a comparably soft fluoropolymer shell are prepared. Application of the melt-shear organization technique yield free-standing fluoropolymer opal and inverse films with remarkably distinct reflection colors and hydrophobic properties. Moreover, these novel opal and inverse opal films were investigated with respect to their optical properties, swelling capability in water, and chemical resistance toward acids and bases.

## 2. Results and Discussion

The following chapter is divided into six sections, starting with the design of fluorine-containing core/shell particles, followed by the preparation of opal and inverse opal films based on these particles by application of the melt-shear organization technique. Finally, the feasibility of herein investigated fluorine-containing opals and inverse opals will be elucidated with respect to their optical properties, chemical resistance, and solvent-induced structural color changes.

### 2.1. Bottom-Up Fabrication of Fluorine-Containing Opal and Inverse Opal Films

**Figure 1:**
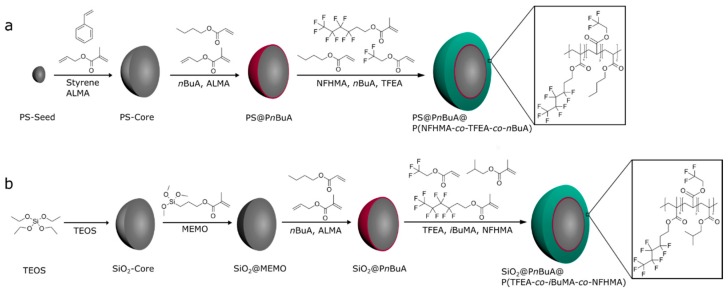
(**a**) Stepwise synthesis of organic fluorine-containing PS@P(NFHMA-*co*-TFEA-*co*-*n*BuA) core/interlayer/shell particles; (**b**) stepwise synthesis of inorganic/organic fluorine-containing SiO2@P(TFEA-*co*-NFHMA-*co*-*i*BuMa) core/interlayer/shell particles. Abbreviations: polystyrene (PS), allylmethacrylate (ALMA), *n*-butylacrylate (*n*BuA), 1*H*,1*H*,2*H*,2*H*-nonafluorohexylmethacrylate (NFHMA), trifluoroethylacrylate (TFEA), tetraethoxysilane (TEOS), 3-methacryloxypropyltrimethoxysilane (MEMO), and *i*-butylmethacrylate (*i*BuMA).

For the preparation of fluorine-containing opal films as well as inverse opal films the tailored design of monodisperse core interlayer shell particles is a basic prerequisite. For this purpose, the complex particle architecture was developed by a step-wise emulsion polymerization as given in Figure 1. In the case of filled opal films (Figure 1a), the hard organic core particles were generated by the polymerization of styrene, combined with the cross-linker butandiol diacrylate (BDDA), followed by a starved feed addition of an emulsion consisting of styrene and allylmethacrylate (ALMA). The particle sizes were analyzed by means of dynamic light scattering (DLS) and transmission electron microscopy (TEM), as described in detail in the following. For the next synthesis step, an additional cross-linked interlayer consisting of *n*-butylacrylate (*n*BuA) and ALMA was introduced. Herein, the residual allyl-moieties of ALMA were capable of acting as grafting anchors for the soft shell material. As already mentioned in the introduction, this step is important to ensure preservation of the spherical shape of the particle cores during processing and during the application of mechanical stress during the melt-shear-organization [27,46,47]. The major part of the outer soft shell consisted of poly(1*H*,1*H*,2*H*,2*H*-nonafluorohexylmethacrylate) (PNFHMA) (60 wt%) as a fluorine-containing polymer. For maintaining a soft and processable polymer mass for the intended extrusion step, a softer comonomer was additionally introduced as shell material. For this purpose, *n*BuA (20 wt%) and trifluoroethylacrylate (TFEA) (20 wt%) as comonomers were copolymerized with an NFHMA monomer. The intermediate glass transition temperature (T*_g_*) decreased up to 14 °C for the copolymer, compared to the respective T*_g_* of the respective PNFHMA homopolymers (T*_g_* = 25 °C) (Figure S1 of the Supporting Information) [48]. These three monomers were used for the formation of the—compared to the hard core material—soft and functional fluorine-containing particle shell material. The successful formation of the core/interlayer/shell-particles was followed by DLS measurements (Figure S2a of the Supporting Information), proving an increase in the diameter after every synthesis step. In detail, the average hydrodynamic diameter of the particles were 206 ± 2 nm for the PS core particles, 211 ± 2 nm for the core/interlayer, and 246 ± 1 nm for the final fluorine-containing core/interlayer/shell particles. It can be concluded from these results that rather monodisperse particles were accessible, which will be important for the optical properties of the opal films (see next sections). The fluorine-containing particles were additionally investigated by using transmission electron microscopy (TEM) (Figure S2b–d), confirming the monodisperse and spherical character of the fluoropolymer-containing core/shell particles. These findings were essential for the fabrication of soft colloid crystal films as described in the next section. Both the DLS and TEM measurements revealed the successful formation of the core/interlayer/shell architecture and sizes of each particles were found to be in excellent agreement with expectations based on monomer consumption and the recipe for emulsion polymerization (*cf*. Experimental Section).

**Figure 2:**
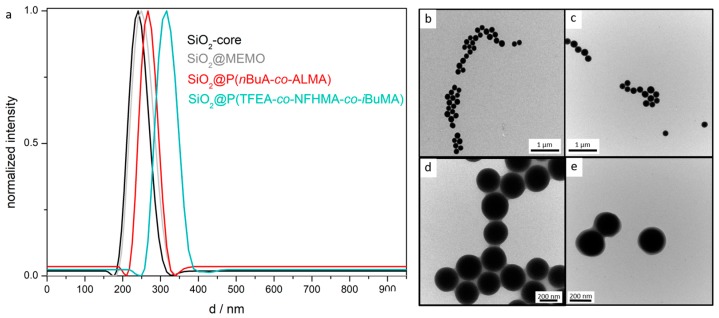
(**a**) DLS measurements of SiO_2_ core particles, MEMO-functionalized SiO_2_ particles, SiO_2_ core/P(*n*BuA-*co*-AMLA)-interlayer particles, and SiO_2_ core/P(*n*BuA-*co*-ALMA)interlayer/P(TFEA-*co*-NFHMA-*co*-*i*BuMA)-shell particles; (**b**) TEM image of SiO_2_-core particles; (**c**) TEM image of MEMO-functionalized SiO_2_ particles; (**d**) TEM image of SiO_2_ core/interlayer particles; (**e**) TEM image of SiO_2_ core interlayer shell particles.

Core/interlayer/shell particles were also synthesized for the preparation of the corresponding inverse opal films. For this purpose, silica (SiO_2_) particles were used as hard core particles. Pristine SiO_2_-particles were synthesized according to the literature by van Blaaderen et al. [49], applying a sol-gel process (Stöber process). For the preparation of monodisperse SiO_2_ particles, tetraethoxysilane (TEOS) in ethanol was used as a precursor, followed by a functionalization of the particle surface with 3-methacryloxypropyltrimethoxysilane (MEMO) [39]. For transferring the particles into the emulsion polymerization, ethanol was substituted by deionized water by azeotropic distillation. In the next step, the MEMO-functionalized SiO_2_-particles were used for the preparation of the interlayer, consisting of poly(*n*BuA-*co*-ALMA). Finally, the polymerization of the outer shell was performed by copolymerizing TFEA (60 wt%), NFHMA (20 wt%), and *iso*-butylmethacrylate (*i*BMA) (20 wt%) as monomers. These inorganic core particles featuring an organic interlayer/shell polymer were examined with respect to morphology and average size by DLS measurements and TEM (Figure 2). All data on the different particles are compiled in Table 1.

In summary, the DLS and TEM measurements again evidenced the successful preparation of well-defined spherical particles and a continuously increasing particle size for each synthesis step. While the particles in Figure 2b,c were still clearly separated from each other, the particles in Figure 2d,e are obviously connected by a soft polymer shell. DLS measurements in general give a larger diameter for particle systems compared to the TEM images, because the hydrodynamic diameters of the particles is determined, which is larger compared to particles in the dried state. For gaining insights into the composition and the thermal properties of the designed functional particles comprising the PS core, the PS@P(NFHMA-*co*-TFEA-*co*-*n*BuA) as well as the SiO_2_@P(TFEA-*co*-NFHMA-*co*-*i*BuMA) core/interlayer/shell particles, differential scanning calorimetry (DSC) measurements were performed. The measured glass transition temperature of the (NFHMA-*co*-TFEA-*co*-*n*BuA)-polymer shell was found to be 14 °C, which was significantly lower than the T*_g_* value found for the PS core, i.e., 100 °C (Figure S1). Moreover, the T*_g_* value of the shell was found to be in between the T*_g_*s of the pure components, i.e., 25 °C for PNFHMA [48], −54 °C for P*n*BuA [50], and −10 °C for PTFEA, confirming the successful copolymerization of the corresponding monomers.

Compared to this organic particle system, the glass transition temperature for the SiO_2_@P(TFEA-*co*-NFHMA-*co*-*i*BuMA) core/shell particles was found to be 27 °C (Figure S3) due to the content of P*i*BuMA, which featured a glass transition temperature of 53 °C for the homopolymers [50]. Hence, successful copolymerization of the respective monomers was evidenced by the presence of the intermediate glass transition temperature. Moreover, these moderate values for the glass transition temperatures should enable processing by means of melt-shear organization of the core/shell particles, which will be described in the following.

**Figure 3:**
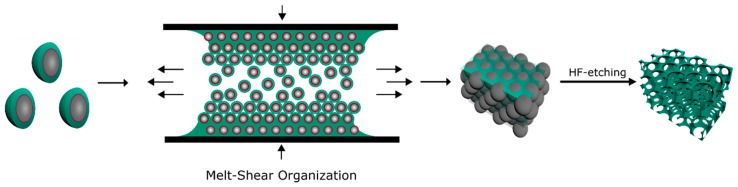
Fabrication of opal films using the melt-shear organization technique and preparation of an inverse opal by HF etching of the SiO_2_ core particles.

For preparation of the elastomeric opal films using the melt-shear organization technique, the core/interlayer/shell particles were precipitated from their dispersion followed by drying at 40 °C. For homogenization of the obtained particle mass as well as for the addition of UV-cross-linking reagents (Irgacure 184, benzophenone, and 1,4-butanedioldiacrylate (BDDA)), the sticky particle mass was mixed using a microextruder at 90 °C (see Experimental Section). During this step, the addition of cross-linking reagents is important for subsequent UV-induced cross-linking reaction of the opal film. For this reason, cross-linking reagents that do not initiate chemical reactions during the melt-shear organization process but can initiate a posteriori are necessary. A cross-linked network, generated by the UV irradiation of the opal films, enhances the mechanical—and therefore the optical—properties of the opal films [46,47,51]. In order to additionally enhance the reflection colors of the opal films, 0.05 wt% carbon black (special black 4, Degussa) powder as an absorber was added during the extrusion. In general, carbon black powder has been found to dramatically enhance the perceived reflection color due to spectrally resonant scattering inside the opal structure without affecting the lattice quality [52]. Moreover, because of the small amount of added carbon black, it is not expected that the refractive index of the fluoropolymer-containing matrix material will be significantly increased. In the next step, the extruded polymer strands were subjected to the melt-shear organization process (Figure 3), allowing the core/shell particles to merge into a colloid crystal structure.

For this purpose, the particle mass was placed between the plates of a press followed by increasing the temperature and applying a pressure up to 100 bar (*cf*. Experimental Section). Within this film formation step, the soft shells generated a continuous matrix embedding the hexagonally arranged hard core particles. The latter formed the final colloidal crystal structure. In order to gain elastomeric properties and to maintain the stability upon etching for the preparation of an inverse opal film, the opal film was irradiated with UV-light to initiate the cross-linking reaction inside the polymer matrix. In this way, an elastomeric fluorine-containing opal film with iridescent reflection colors was prepared as studied in detail in the next section.

### 2.2. Optical Properties and Morphology of the PS@P(NFHMA-co-TFEA-co-nBuA) Opal Film

For the preparation of the elastomeric fluorine-containing opal films, some basic requirements must be fulfilled: (i) a periodic close-packed arrangement of the hard spheres, (ii) the monodispersity of the core particles, and (iii) a refractive index contrast between the hard core, respectively the voids, and the matrix material.

According to Bragg´s law of diffraction combined with Snell´s law (Equation (1)), the perceived wavelength of reflection (λ_111_) is influenced by the average refractive index *n_eff_*, which can be calculated by considering the refractive index *n_i_* and the volume fractions ϕ*_i_* for all ingredients of the opal (Equation (2)). The wavelength of reflection depends also on the periodicity α_111_ and the angle of incident light θ [24].

(1) $$\lambda_{111}=2\alpha_{111}{\left(n_{eff}^{2}-\sin^{2}\theta \right)}^{1/2}$$

(2) $$n_{eff}=\sum n_{i}\phi_{i}$$

**Figure 4:**
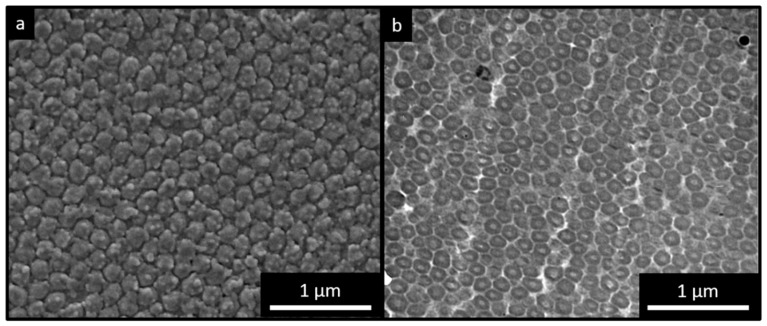
(**a**) SEM image: surface of PS@P(NFHMA-*co*-TFEA-*co*-*n*BuA) opal film; (**b**) TEM image: ultra-thin cut of the cross-section of a PS@P(NFHMA-*co*-TFEA-*co*-*n*BuA) opal film.

For this purpose, an organic core-particle consisting of polystyrene (*n_eff_* ≅ 1.59) [53] and a shell material containing a high content of poly(NFHMA) (*n_eff_* ≅ 1.35) [54] was used. Compared to previously reported organic core/shell opal films, this combination featured a higher refractive index contrast (Δ*n_eff_* ≤ 0,24). From the photographs obtained by scanning electron microscopy (SEM) (Figure 4a), it can be concluded that the particle-based film consists of closely packed and hexagonally aligned layers of particles. Additional to these findings, transmission electron microscopy (TEM) images obtained for ultra-thin slices prepared from the opal film are shown in Figure 4b, revealing the individual PS particles embedded in a lighter appearing matrix. The lighter matrix corresponded to the fluoropolymer shell material, having less electron contrast compared to the aromatic PS core particles. However, in the case of the TEM images, the core particles appeared to be more distorted. This can be explained by the fact that the spherical domains having a size of 192 ± 10 nm are much larger than the microtomed thin slices, which are approximately 50–70 nm in thickness. During ultramicrotoming of the bulk polymer films with spherical domains inside a soft matrix, the probability for perfect cutting of spherical objects is reduced, which is a known problem for spherical domains during sample preparation by using ultramicrotomy [55].

**Figure 5:**
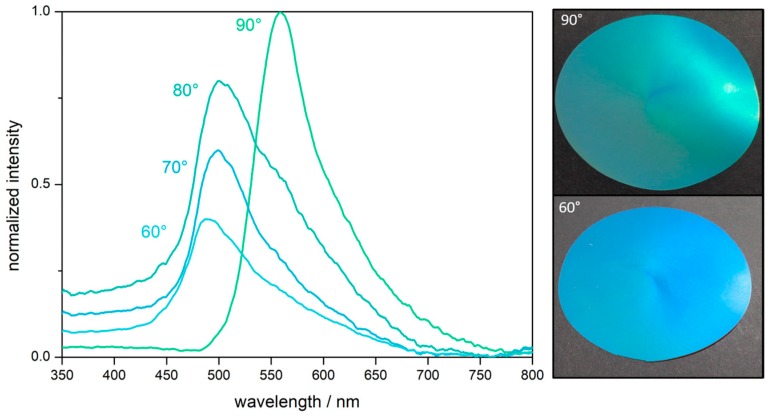
Angle-dependent UV/Vis reflection spectra of the opal film prepared from PS@P(NFHMA-*co*-TFEA-*co*-*n*BuA) core/interlayer/shell particles and corresponding photographs of the opal film at an angle of view of 90° and 60°. The final opal discs featured a diameter of 8 cm.

To determine the position of the Bragg peak and evidencing a good optical performance based on the ordered particles, angle-dependent UV/Vis measurements were performed (Figure 5). The measurements were recorded for angles of incident between 90° and 60°. Within the corresponding spectra, a distinct Bragg peak at a wavelength of 558 nm was observed. At lower angles of incidence, the value for the reflected light, i.e., the Bragg peak was blue-shifted from 558 nm at 90° to 486 nm at 60° according to Bragg´s law of diffraction. This finding proved the existence of a structural color for the herein designed fluorine-containing opal films. Moreover, it can be concluded that the melt-shear organization of the PS@P(NFHMA-*co*-TFEA-*co*-*n*BuA) particles leads to a regularly ordered particle-based film with brilliant reflection colors.

### 2.3. Chemical Resistance of the PS@P(NFHMA-co-TFEA-co-nBuA) Opal Film

In general, polymers having a high content of fluorine-containing monomers feature a good chemical resistance toward acids or bases [56]. In order to investigate the chemical resistance, the here prepared opal films were exposed to a strong acid (hydrochloric acid pH = 1) and base (potassium hydroxide pH = 13), followed by subsequent UV/Vis measurements of the samples. In Figure S4, UV/Vis spectroscopy measurements of the opal films treated with potassium hydroxide solution and hydrochloric acid are shown. The untreated film featured a reflection color maximum at a wavelength of approximately 525 nm. When the fluorine-containing opal film was treated with deionized water, the reflection peak of the opal film slightly shifted to 544 nm, which was due to the swelling capability of the matrix material. However, treatment of the swollen opal films with concentrated acid or concentrated base did not lead to a significant change of the optical properties. In more detail, the reflection color of the opal film treated for 20 min with potassium hydroxide was shifted only toward 6 nm and Bragg peak maximum was finally located at a wavelength of 550 nm at an angle of view of 90°. When the opal film was treated for 20 min with hydrochloric acid, the peak was located at a wavelength of 540 nm, i.e., only with a shift of 4 nm. Therefore, UV/Vis measurements proved the excellent order and resistance of the opal films under harsh conditions.

### 2.4. Hydrophobicity and Solvent Response of the PS@P(NFHMA-co-TFEA-co-nBuA) Opal Film

**Figure 6:**
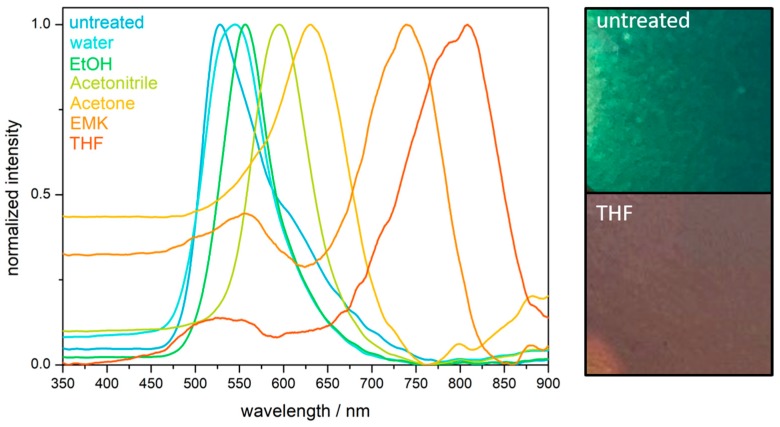
UV/Vis reflection spectra of a solvent-treated opal film based on PS@P(NFHMA-*co*-TFEA-*co*-*n*BuA) core/interlayer/shell particles (**left**) and exemplary photographs of the opal film prior to and after treatment with THF (**right**). Abbreviations: ethanol (EtOH), ethyl methyl-ketone (EMK), and tetrahydrofurane (THF).

The hydrophobic character of the fluorine-containing opals was determined by contact angle measurements. A contact angle between the opal film surface and a sessile drop of deionized water of 106 ± 3° was obtained, which categorized the surface hydrophobic (see Figure S5a). As described in the introduction, fluoropolymers have already found an extensive application as a low surface energy material for water repellency applications [37,57]. In general, the wettability of surfaces with liquid depends on two factors: (i) the chemical factor based on the low surface energy and (ii) the geometrical factors mainly given by roughness and tailored architecture of the surface. As can be concluded from the SEM image in Figure 4a, the surface of the opal film is not exceptional rough or structured, and the water repellency effect is therefore considered to stem from the chemical factor, i.e., the used fluorine-containing polymers. Despite the high chemical resistance and water repellency, the domain sizes of the opal film could be influenced upon treatment with various organic solvents. When the distance between the spheres forming the colloidal crystal stack is in the range of half the wavelength of visible light, structural colors can be observed, as described by Bragg´s law of diffraction. Solvent treatment caused a swelling-induced volume change of the matrix polymer, resulting in the increase of surface plane spacing of the closely packed PS spheres embedded in the cross-linked opal film. Moreover, the Bragg conditions for the respective colors was also influenced by the refractive index of the solvent used for matrix swelling, which has been described in earlier works [23,24,58]. Typically, the swelling behavior of the matrix materials leads to a shift of the reflection peak maximum toward a higher wavelength. In other words, the volume expansion of the polymer matrix induced by solvent treatment leads to a red-shift of the reflection peak maxima. Exemplarily, the peak maximum shifted from 525 nm for the untreated opal film to 808 nm after treatment with THF (Figure 6). Depending on the swelling capability, the used media (water, ethanol, acetonitrile, acetone, ethyl methyl ketone (EMK), and THF) will lead to a more pronounced reflection peak maximum shift within this order. Noteworthy, after complete evaporation of the used solvents, the fluorine-containing opals reached the original peak maximum value after repeated solvent treatment-evaporation cycles, at least three times.

In summary, the convenient preparation of fluoropolymer-containing opal films featuring angle-dependent reflection colors and solvent-responsive behavior were prepared. Moreover, the reflection peak position of the fluorine-containing opal films were not altered by treatment with concentrated acids and bases.

### 2.5. Optical and Structural Properties of the SiO_2_@P(TFEA-co-NFHMA-co-iBuMA) Inverse Opal Film

**Figure 7:**
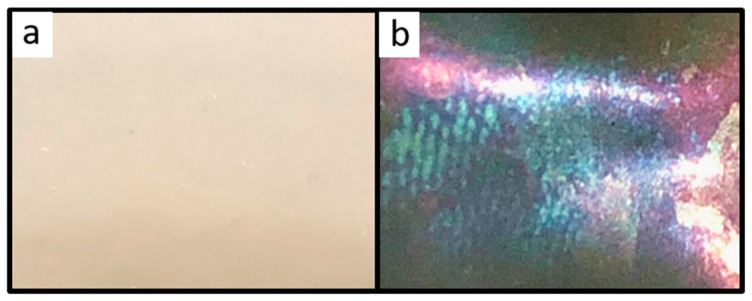
(**a**) Photographic picture of the filled opal film based on SiO_2_@P(TFEA-*co*-NFHMA-*co*-*i*BuMA) core/interlayer/shell particles, without reflection colors; (**b**) photographic picture of the inverse opal film based on SiO_2_@P(TFEA-*co*-NFHMA-*co*-*i*BuMA) core/interlayer/shell particles, with reflection colors. The cutout of the inverse opal discs featured a diameter of 2 cm.

For the preparation of fluoropolymer inverse opal films, SiO_2_ core particles with a comparably soft fluorine-copolymer shell were subjected to the melt-shear-organization technique. The silica-core particles were etched by HF treatment in a subsequent step in order to gain access to the inverted opal structure (see Experimental Section). The opal films prior to the etching process featured no bright reflection color, since the refractive index contrast Δ*n_eff_* between the silica core particles (*n_eff_* = 1.43) [59] and the fluoropolymer matrix (*n_eff_* ≤ 1.41) was rather low [57]. However, after removal of the silica core particles, the ordered pores inside the fluoropolymer matrix led to a more pronounced refractive index contrast and therefore to the appearance of iridescent structural colors. Figure 7a shows photographs of the opal film filled with SiO_2_ cores featuring no reflection colors, while in Figure 7b the inverted opal film is given showing structural colors.

**Figure 8:**
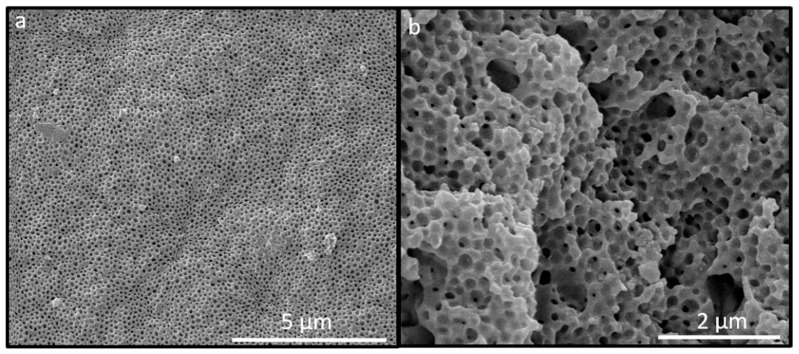
SEM photograph of the topography (**a**) and corresponding cross-section SEM photograph (**b**) of an inverse opal film based on SiO_2_@P(TFEA-*co*-NFHMA-*co*-*i*BuMA) core/interlayer/shell particles after melt-shear organization and subsequent HF etching.

In order to obtain more intensive reflection colors, a higher content of pores was of utmost importance. Therefore, the film was treated with hydrofluoric acid for full removal of the SiO_2_ cores after several days. Corresponding SEM studies revealed that the surface of the inverse fluoropolymer opal film is open-porous with a uniform diameter of the pores of 175 ± 5 nm (Figure 8a). The corresponding cross-section (Figure 8b) revealed that the SiO_2_ core particles were also removed in the interior of the opal film. It has to be mentioned that the pore order and pore size distribution seemed to be influenced with respect to their spherical shape within the SEM photographs for the cross-section compared to the film topography. This might be caused by sample preparation using freeze-fracturing for the comparably soft inverse opal films. Moreover, for the preparation of the fluoropolymer inverse opal films, a slightly different polymer composition was chosen. In comparison to the opal films described in Section 2.2, a lower fraction of NFHMA (20 wt%) and a higher amount of TFEA (60 wt%) was used. The reason for changing the composition and for additionally introducing *i*BuMA (20 wt%) instead of *n*BuA was the softness of the final inverse opal film leading to a pore collapse after the etching process. While using a fluropolymer copolymer with P*i*BMA, the glass transition temperature could be increased to 25 °C, suitable for convenient core/shell particle processing and for the preparation of a free-standing inverse opal film. The T*_g_* was determined to be 25 °C, as studied by DSC measurements (Figure S3).

**Figure 9:**
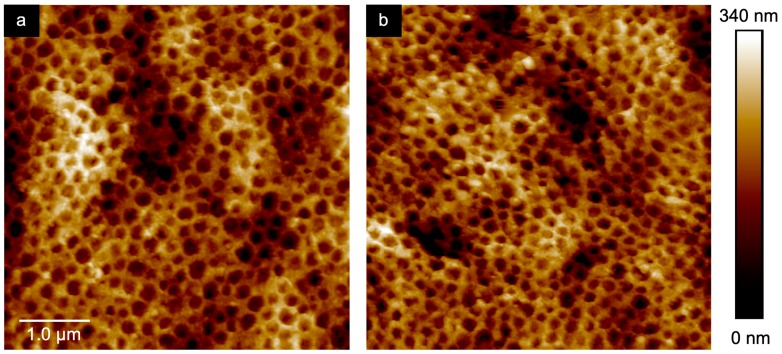
AFM image of the dried inverse opal film obtained after etching of the SiO_2_@P(TFEA-*co*-NFHMA-*co*-*i*BuMA)-based particle films (**a**) and the same film measured in water (**b**) Scale bars correspond to 1 µm (see text).

To further investigate the pore order and to visualize influence on the morphology upon water treatment, atomic force microscopy (AFM) studies were additionally carried out. Corresponding AFM measurements for the dried film and for the inverse opal film in water are given in Figure 9. In good accordance with the SEM images in Figure 8, AFM studies confirmed the presence of well-ordered hexagonally aligned pores. The average pore size in the dried state was determined to be 179 ± 8 nm, which was in good agreement with the pore size determined in the corresponding SEM image (175 ± 5 nm). Figure 9b give the results for AFM measurement of the same inverse opal film in water. As a finding, the average pore size was found to 153 ± 10 nm in diameter, which reflects a slight swelling capability of the water-treated inverse opal matrix material.

### 2.6. Chemical Resistance and Solvent-Responsivness of the Inverse Opal Film

**Figure 10:**
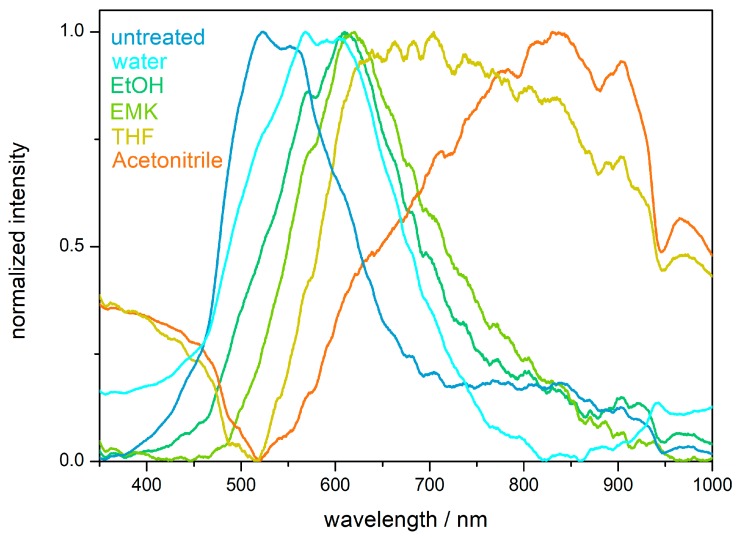
UV/Vis reflection spectra of solvent-treated inverse opal film based on P(TFEA-*co*-NFHMA-*co*-*i*BuMA). Abbreviations: ethanol (EtOH), ethyl methyl ketone (EMK), and tetrahydrofuran (THF).

As already mentioned in the previous section, the hydrophobic character of the fluoropolymer-based opal materials was proven by contact angle measurements. Here, contact angle measurements for the inverse opal film derived after etching of SiO_2_@P(TFEA-*co*-NFHMA-*co*-*i*BuMA)-based particle films, resulted in a contact angle of 102 ± 2° (Figure S5b). Along with the results obtained from the AFM measurements, a slight swelling of the inverse opal film upon water treatment was observed, which also led to a shift of the maximum peak position during UV/Vis spectroscopy measurements (Figure 10). In detail, the dried inverse opal film featured a reflection color peak at a wavelength of 518 nm, while the peak shifted to a wavelength of 570 nm upon water treatment. This was found to be a remarkable difference compared to the results obtained for the filled opals as shown in Figure 6, revealing a peak maximum shift of only 16 nm. This finding underpins the more pronounced sensing capability for water (and other media) for the inverse opal structures compared to the filled opal films. This effect is even more pronounced while using organic solvents (Figure 10). The peak maxima shifted in polar media, such as water (peak maximum at 570 nm) or ethanol (612 nm), tetrahydrofuran (704 nm), and acetonitrile (836 nm), respectively. This observation can be explained by the swelling capability induced by the different solvents for the fluorine-containing matrix. In contrast, the appearance of the structural colors is only slightly affected by the change of the refractive index contrast, since the different refractive indices of the used solvents are similar, i.e., 1.33 for water, 1.36 for ethanol, 1.37 for ethyl methyl ketone, 1.40 for THF, and 1.34 for acetonitrile [60,61].

Finally, the chemical resistance of the inverse opal film caused by fluorine-containing matrix was also examined by treatment with a strong acid (hydrochloric acid pH = 1) and base (potassium hydroxide pH = 13) by UV/Vis spectroscopy measurements (Figure S6). Despite the slightly reduced fluorine-content inside the inverse opal matrix, only a small shift of the reflection peak maximum of approximately 10 nm was obtained from the corresponding spectra. This finding proved the high chemical resistance also for the inverse opal films upon potassium hydroxide and hydrochloric acid treatment.

## 3. Experimental

### 3.1. Materials and Methods

1*H*,1*H*,2*H*,2*H*-Nonafluorohxylmethacrylate (NFHMA, 95%) was obtained from ABCR (Karlsruhe, Germany), trifluoroethylacrylat (TFEA, >98%) from TCI (Eschborn, Germany), Butandioldiacrylate (BDDA), and Irgacure 184 from BASF (Ludwigshafen, Germany). Dowfax 2A1, a surfactant having a dual polar head group and a non-polar alkyl chain was obtained from Dow Chemicals (Midland, MI, USA), carbon black (Special Black 4) by Degussa GmbH (Essen, Germany), and benzophenon, the UV-initiator, was donated by Merck Chemicals (Darmstadt, Germany). All other chemicals were purchased from VWR (Radnor, PA, USA), Acros Organics, Fisher Scientific (Schwerte, Germany), and Sigma Aldrich (St. Louis, MO, USA) and used as received, if not otherwise mentioned. Prior to use for the polymerization, inhibitors were removed from the monomers *n*BuA, *i*BuMA, and styrene by passing through an alumina column (basic, 50–200 µm, Acros Organics).

Dynamic light scattering (DLS) measurements of the particle dispersions were performed with a Zetasizer ZS90 (Malvern Instruments, Malvern, UK). The measurements were carried out at 25 °C at an angle of 90°. For the evaluations, the z-weight average hydrodynamic diameter was used. Transmission electron microscopy (TEM) was realized on a Zeiss EM 10 electron microscope (Oberkochen, Germany) operating at 60 kV. The images were recorded in bright field mode with a slow scan CCD camera obtained from TRS Tröndle (Morrenweis, Germany). The control of the camera was computer-assisted using ImageSP from TRS. Scanning electron microscopy (SEM) was performed on a Philips XL30 FEG (Amsterdam, Netherlands) with an operating voltage of 5–10 kV. The samples were previously coated for 100 s at 30 mA with a thin gold layer, using a Quorum Q300T D sputter coater (Lewes, UK). Angle dependent reflection measurements were performed using a custom built goniometer setup measured in steps of 10° of scattering angle. All other reflection spectra were recorded using a Vis/-NIR fiber spectrometer USB 2000, Ocean Optics (Ostfildern, Germany). For the measurements, a deuterium/tungsten halogen lamp DT mini 2, Ocean Optics was used. Measurements in water or solvents were carried out at normal light incidence (90°). Thermal properties of the core interlayer shell particles were characterized using a differential scanning calorimeter (DSC) from Mettler Toledo (Columbus, OH, USA) DSC-1 in the temperature range from −50 °C to 150 °C with a heating rate of 20 K min^−1^ in a nitrogen atmosphere. Atomic force microscopy measurements were accomplished in the PeakForce Tapping mode with an Icon Dimension Bruker AXS (Santa Barbara, CA, USA). Images with dimensions of 5 × 5 µm^2^ (512 × 512 pixel) were taken in air and deionized water at a scan rate of 1 Hz using a maximum force of 2 nN. The inverse optical sensitivity of the laser detection system was calibrated by pushing the cantilever with the tip onto a stiff substrate (sapphire) and relating the deflection of the laser spot on the photosensitive diode with the movement of the z-piezo. The cantilever spring constant was 0.8 nN/nm measured by the thermal noise method [62]. The contact angle (CA) was measured using the sessile-drop-method with a Contact angle system DATAPhysics OCA 15 EC (Filderstadt, Germany) using 2 µL droplets of deionized water. The measurements were conduced in a controlled climatic chamber at a temperature of 23 ± 2 °C and a relative humidity of 40%. Contact angles were determined geometrically using the SCA20 software by aligning a tangent from the surficial contact point along the droplet profile.

### 3.2. Synthesis of PS@PBuA@P(NFHMA-co-TFEA-co-nBuA) Core/Interlayer/Shell-Particles

The stepwise generation of fluorine-containing polymer based particles is illustrated in Figure 1a. The corresponding monodisperse PS particles were synthesized according to starved feed emulsion polymerization protocols. A 1 L reactor under an argon stream, equipped with a reflux condenser and a stirrer, was heated up to 80 °C and filled with a dispersion of 3.6 g of styrene, 0.4 g of BDDA, 0.1 g of SDS, and 280 g of degassed water. The polymerization was initiated by adding solutions of 70 mg of sodiummetabisulfite (SBS) in 5 g of water and 500 mg of sodiumperoxodisulfate (SPS) in 5 g of water. After 20 min, a monomer emulsion containing 3.5 g of ALMA, 31.5 g of styrene, 180 mg of KOH, 164 mg of SDS 105 mg of Igepal, and 40.9 g of water was added continuously with a flow rate of 0.5 mL/min. After one hour of reaction time, the polystyrene particles with an average diameter of 206 nm were characterized and stored for further use.

The core-shell particle featuring a copolymer of P*n*BuA, PNFHMA, and PTFEA as a soft shell was synthesized in a 100 mL double-wall reactor equipped with a stirrer and reflux condenser in an argon atmosphere at 80 °C. For this purpose, 60 g of the PS-particle dispersion with a solid content of 8.4 wt% was filled into the reactor, and the emulsion polymerization was initiated by the addition of 14 mg of SBS and 77 mg of SPS dissolved in 2 mL of deionized water. After 20 min reaction time, a monomer emulsion (ME1) consisting of 75 mg of ALMA, 425 mg of *n*BuA, 10 mg of Dowfax 2A1, 5 mg of SDS, and 2.5 g of deionized water was added with a flow rate of 0.2 mL min^−1^ using a rotary piston pump. After the complete addition of ME1, a solution of 12 mg of SPS in 2 g of water was added and a second monomer emulsion (ME2) consisting of 2.7 g of NFHMA, 0.9 g of TFEA, 0.9 g of *n*BuA, 45 mg of KOH, 16 mg of SDS, 13 mg of Dowfax 2A1, and 5.22 g of water were continuously added with a flow rate of 0.2 mL min^−1^. After complete addition of the ME2, the temperature of polymerization was maintained for an additional hour prior to cooling the vessel to room temperature. The average particle diameter of the core shell particle was determined to be 246 ± 1 nm (DLS).

### 3.3. Synthesis of SiO_2_@PBuA@P(TFEA-co-NFHMA-co-iBuMA) Core/Interlayer/Shell-Particles

Silica particle dispersion with a solid content of 2.5 wt% in ethanol were prepared using a sol-gel process (Stöber process) according to the protocol described by van Blaaderen [49]. A mixture of 1.3 L of ethanolic silica dispersion with 1.6 mL of 3-methacryloxypropyltrimethoxysilane (MEMO) was heated to 60 °C and stirred for 2 h. After functionalization, ammonia was carefully removed by azeotropic distillation at 60 °C under reduced pressure, while the volume was kept constant by tcontinuous addition of ethanol. When the dispersion was free of ammonia, the volume was reduced to 300 mL at 60 °C. For transferring the silica particle dispersion into an aqueous medium for the intended emulsion polymerization, a solution of 50 mg of SDS in 100 mL of deionized water was added, and ethanol was removed by azeotropic distillation. The volume was kept constant by continuous addition of water. The final MEMO-functionalized aqueous dispersion featured a silica solid content of 8.83 wt%. The average particle diameter of the particles after functionalization was 249 ± 4 nm, as determined by DLS measurements.

For synthesis of the hybrid core-shell particles, 70 g of the pristine SiO_2_ particle dispersion was filled in a 100 mL double wall reactor. The emulsion polymerization was initiated at 80 °C by the addition of a solution of 16 mg of SBS and 94 mg of SPS in 2 mL of deionized water. After 20 min, a monomer emulsion (ME1) consisting of 83 mg of ALMA, 0.47 g of *n*BuA, 11 mg of Dowfax 2A1, 6 mg of SDS, and 2.77 g of water were added by a rotary piston pump with a flow rate of 0.2 mL min^−1^. After a 15 min reaction time, 15 mg of SPS in 2 mL of water was added and another monomer emulsion (ME2) composed of 3.55 g of TFEA, 0.9 g of NFHMA, 0,9 g of *i*BuMA, 7 mg of KOH, 16 mg of SDS, 12 mg of Dowfax 2A1, and 5.2 g of water was continuously added with a flow rate of 0.2 mL min^−1^. After an additional hour, the product was cooled to room temperature. The diameter of the core/shell particle was 315 ± 9 nm, as determined by DLS measurements.

### 3.4. Particle Processing and Preparation of Opal and Inverse Opal Films

For the preparation of elastomeric opal films, the obtained PS@P*n*BuA@P(NFHMA-*co*-TFEA-*co*-*n*BuA) particles were precipitated in methanol containing 20% of saturated sodium chloride solution. The precipitate was filtered, washed with water, and dried under vacuum at 40 °C. For homogenization, the precursor powder was mixed with 7.25 wt% BDDA, 1 wt% benzophenone, 1 wt% Irgacure 184, and 0.05 wt% carbon black (special black 4, Degussa) in a microextruder (HAAKE Minilab II350, Thermo Scientific, Waltham, MA, USA) at 90 °C. For opal film formation, a 2 g portion of the polymeric mass was covered by two PET foils and heated between two steel-plates in a Collin laboratory press (Dr. Collin GmbH, Ebersberg, Germany). The particle mass was transduced into an opal disc film of approximately 8 cm in size by using the melt-shear organization technique at 90 °C and 100 bar. Subsequently, the opal films were irradiated with a mercury lamb (UVA Cube 2000, Dr Hoenle AG, Gräfelfing, Germany) with an output power of 1000 W at a distance of 10 cm for 3 min at both sides, for cross-linking reactions. For conversion of the opal film containing SiO_2_ core particles into an inverse opal film, the cores were removed by etching with hydrofluoric acid (HF, 10 wt% in water) for 4 days. The films were washed with plenty of deionized water several times.

## 4. Conclusions

In conclusion, the current work demonstrated an efficient protocol for preparation of fluorine-containing core/interlayer/shell particles. For the hard core particle preparation, inorganic SiO_2_ or PS particles were used, whereas the soft shell was formed by a stepwise emulsion polymerization of highly fluorinated monomers, i.e., 1*H*,1*H*,2*H*,2*H*-nonafluorohexylmethacrylate (NFHMA) and trifluoroethylacrylate (TFEA), leading to well-defined fluorine-containing core/shell particles. The melt-shear organization technique was applied for the preparation of easily scalable fluoropolymer opal disc films. The opal film based on SiO_2_ core particles were subjected to an etching protocol using hydrofluoric acid to gain access to fluoropolymer inverse opal structures. Characterization of the particles, opal films, and inverse opal films was carried out using DLS, TEM, SEM, AFM, DSC, UV/Vis spectroscopy, and contact angle measurements, evidencing the size, uniformity, and order after the melt-shear organization and subsequent etching. The feasibility of fluorine-containing polymers as functional matrix materials, leading to opal films featuring brilliant reflection colors, was shown by the good refractive index contrast compared to the PS core material (or air voids in the case of the inverse opals). Finally, swelling capability and stimuli-responsiveness upon treatment with different solvents were investigated, and the reversible switching behavior of the optical properties was shown. Moreover, the inverse opals revealed good hydrophobic properties and excellent chemical resistance toward strong acids and bases. Along with the good structural colors, we envisage the herein investigated fluorine-containing opal and inverse opal films as promising candidates in the field of robust coatings and switchable optical sensing devices.

## Figures and Tables

**Table 1:** Average hydrodynamic diameter of particles measured by means of DLS and average diameters of the dried particle, as determined by TEM. In the case of TEM analysis, 50 particles were measured with respect to their size.

Particle	DLS (d/nm)	TEM (d/nm)
PS	206 ± 2	192 ± 10
PS@P(*n*BuA-*co*-ALMA)	211 ± 2	202 ± 6
PS@P(NFHMA-*co*-TFEA-*co*-*n*BuA)	246 ± 1	238 ± 11
SiO_2_	240 ± 1	232 ± 6
SiO_2_@MEMO	249 ± 4	234 ± 11
SiO_2_@P(*n*BuA-*co*-ALMA)	271 ± 1	261 ± 8
SiO_2_@P(TFEA-*co*-NFHMA-*co*-*i*BuMa)	315 ± 9	303 ± 4

## Supplementary Materials

The following information are available online, Figure S1: DSC thermogram of PS based core/shell particles; Figure S2: DLS measurements and TEM images of PS based core/shell particles; Figure S3: DSC thermogram of SiO_2_ based core/shell particles; Figure S4: UV/Vis spectra of an opal film; Figure S5: Photograph of drops of water on opal and invers opal films; Figure S6: UV/Vis spectra of an inverse opal film.

## References

[1] Drobny J.G. (2009). Technology of Fluoropolymers.

[2] Munekata S. (1988). Fluoropolymers as coating material. Progr. Org. Coat..

[3] Smith D.W., Iacono S.T., Iyer S.S. (2014). Handbook of Fluoropolymer Science and Technology.

[4] Cui Z., Drioli E., Lee Y.M. (2014). Recent progress in fluoropolymers for membranes. Progr. Polym. Sci..

[5] Ameduri B., Boutevin B. (2004). Well-Architectured Fluoropolymers: Synthesis, Properties and Applications.

[6] Gardiner J. (2015). Fluoropolymers: Origin, Production, and Industrial and Commercial Applications. Aust. J. Chem..

[7] Moore A.L. (2005). Fluoroelastomers Handbook: The Definitive User’s Guide and Databook.

[8] Dams R., Hintzer K. (2017). Chapter 1 Industrial Aspects of Fluorinated Oligomers and Polymers. Fluorinated Polymers: Volume 2: Applications.

[9] Sun W., Zhou S., You B., Wu L. (2013). A facile method for the fabrication of superhydrophobic films with multiresponsive and reversibly tunable wettability. J. Mater. Chem. A.

[10] Shi F., Song Y., Niu J., Xia X., Wang Z., Zhang X. (2006). Facile Method to Fabricate a Large-Scale Superhydrophobic Surface by Galvanic Cell Reaction. Chem. Mater..

[11] Feng C.L., Zhang Y.J., Jin J., Song Y.L., Xie L.Y., Qu G.R., Jiang L., Zhu D.B. (2001). Reversible Wettability of Photoresponsive Fluorine-Containing Azobenzene Polymer in Langmuir-Blodgett Films. Langmuir.

[12] Pei Y., Travas-Sejdic J., Williams D.E. (2012). Reversible electrochemical switching of polymer brushes grafted onto conducting polymer films. Langmuir.

[13] Hu J., Meng H., Li G., Ibekwe S.I. (2012). A review of stimuli-responsive polymers for smart textile applications. Smart Mater. Struct..

[14] Drelich J., Chibowski E., Meng D.D., Terpilowski K. (2011). Hydrophilic and superhydrophilic surfaces and materials. Soft Matter.

[15] Wenzel R.N. (1936). Resistance of Solid Surfaces to Wetting by Water. Ind. Eng. Chem. Res..

[16] Cassie A.B.D., Baxter S. (1944). Wettability of porous surfaces. Trans. Faraday Soc..

[17] Whitesides G.M. (2005). Nanoscience, nanotechnology, and chemistry. Small.

[18] Piao Y., Burns A., Kim J., Wiesner U., Hyeon T. (2008). Designed Fabrication of Silica-Based Nanostructured Particle Systems for Nanomedicine Applications. Adv. Funct. Mater..

[19] Phillips K.R., Vogel N., Hu Y., Kolle M., Perry C.C., Aizenberg J. (2014). Tunable Anisotropy in Inverse Opals and Emerging Optical Properties. Chem. Mater..

[20] Huang X., Chen J., Lu Z., Yu H., Yan Q., Hng H.H. (2013). Carbon inverse opal entrapped with electrode active nanoparticles as high-performance anode for lithium-ion batteries. Sci. Rep..

[21] Schäfer C.G., Vowinkel S., Hellmann G.P., Herdt T., Contiu C., Schneider J.J., Gallei M. (2014). A polymer based and template-directed approach towards functional multidimensional microstructured organic/inorganic hybrid materials. J. Mater. Chem. C.

[22] Schäfer C.G., Gallei M., Zahn J.T., Engelhardt J., Hellmann G.P., Rehahn M. (2013). Reversible Light-, Thermo-, and Mechano-Responsive Elastomeric Polymer Opal Films. Chem. Mater..

[23] Schäfer C.G., Biesalski M., Hellmann G.P., Rehahn M., Gallei M. (2013). Paper-supported elastomeric opal films for enhanced and reversible solvatochromic response. J. Nanophotonics.

[24] Schäfer C.G., Winter T., Heidt S., Dietz C., Ding T., Baumberg J.J., Gallei M. (2015). Smart polymer inverse-opal photonic crystal films by melt-shear organization for hybrid core–shell architectures. J. Mater. Chem. C.

[25] Schaffner M., England G., Kolle M., Aizenberg J., Vogel N. (2015). Combining Bottom-Up Self-Assembly with Top-Down Microfabrication to Create Hierarchical Inverse Opals with High Structural Order. Small.

[26] Phillips K.R., England G.T., Sunny S., Shirman E., Shirman T., Vogel N., Aizenberg J. (2016). A colloidoscope of colloid-based porous materials and their uses. Chem. Soc. Rev..

[27] Gallei M. (2018). Functional Polymer Opals and Porous Materials by Shear-Induced Assembly of Tailor-Made Particles. Macromol. Rapid Commun..

[28] Schüth F., Schmidt W. (2002). Microporous and Mesoporous Materials. Adv. Mater..

[29] Stein A. (2003). Advances in Microporous and Mesoporous Solids—Highlights of Recent Progress. Adv. Mater..

[30] Thomas A., Goettmann F., Antonietti M. (2008). Hard Templates for Soft Materials: Creating Nanostructured Organic Materials. Chem. Mater..

[31] Llusar M., Sanchez C. (2008). Inorganic and Hybrid Nanofibrous Materials Templated with Organogelators. Chem. Mater..

[32] Joshi R.K., Schneider J.J. (2012). Assembly of one dimensional inorganic nanostructures into functional 2D and 3D architectures. Synthesis, arrangement and functionality. Chem. Soc. Rev..

[33] Scheid D., Cherkashinin G., Ionescu E., Gallei M. (2014). Single-source magnetic nanorattles by using convenient emulsion polymerization protocols. Langmuir.

[34] Galisteo-López J.F., Ibisate M., Sapienza R., Froufe-Pérez L.S., Blanco Á., López C. (2011). Self-assembled photonic structures. Adv. Mater..

[35] Kang H., Lee J.S., Chang W.S., Kim S.H. (2015). Liquid-impermeable inverse opals with invariant photonic bandgap. Adv. Mater..

[36] Vogel N., Belisle R.A., Hatton B., Wong T.S., Aizenberg J. (2013). Transparency and damage tolerance of patternable omniphobic lubricated surfaces based on inverse colloidal monolayers. Nat. Commun..

[37] Wu Y., Zhou S., Wu L. (2016). Fabrication of Robust Hydrophobic and Super-Hydrophobic Polymer Films with Onefold or Dual Inverse Opal Structures. Macromol. Mater. Eng..

[38] Ruhl T., Spahn P., Hellmann G.P. (2003). Artificial opals prepared by melt compression. Polymer.

[39] Finlayson C.E., Baumberg J.J. (2017). Generating Bulk-Scale Ordered Optical Materials Using Shear-Assembly in Viscoelastic Media. Materials.

[40] Scheid D., Stock D., Winter T., Gutmann T., Dietz C., Gallei M. (2016). The pivotal step of nanoparticle functionalization for the preparation of functional and magnetic hybrid opal films. J. Mater. Chem. C.

[41] Winter T., Su X., Hatton T.A., Gallei M. (2018). Ferrocene-Containing Inverse Opals by Melt-Shear Organization of Core/Shell Particles. Macromol. Rapid Commun..

[42] Vowinkel S., Schäfer C.G., Cherkashinin G., Fasel C., Roth F., Liu N., Dietz C., Ionescu E., Gallei M. (2016). 3D-ordered carbon materials by melt-shear organization for tailor-made hybrid core–shell polymer particle architectures. J. Mater. Chem. C.

[43] Vowinkel S., Malz F., Rode K., Gallei M. (2017). Single-source macroporous hybrid materials by melt-shear organization of core-shell particles. J. Mater. Sci..

[44] Vowinkel S., Boehm A., Schäfer T., Gutmann T., Ionescu E., Gallei M. (2018). Preceramic Core-Shell Particles for the Preparation of Hybrid Colloidal Crystal Films by Melt-Shear Organization and Conversion into Porous Ceramics. Mater. Des..

[45] Zhao Q., Finlayson C.E., Snoswell D.R.E., Haines A., Schäfer C., Spahn P., Hellmann G.P., Petukhov A.V., Herrmann L., Burdet P. (2016). Large-scale ordering of nanoparticles using viscoelastic shear processing. Nat. Commun..

[46] Schäfer C.G., Smolin D.A., Hellmann G.P., Gallei M. (2013). Fully Reversible Shape Transition of Soft Spheres in Elastomeric Polymer Opal Films. Langmuir.

[47] Schäfer C.G., Viel B., Hellmann G.P., Rehahn M., Gallei M. (2013). Thermo-cross-linked Elastomeric Opal Films. ACS Appl. Mater. Interfaces.

[48] Jiang J., Zhang G., Wang Q., Zhang Q., Zhan X., Chen F. (2016). Novel Fluorinated Polymers Containing Short Perfluorobutyl Side Chains and Their Super Wetting Performance on Diverse Substrates. ACS Appl. Mater. Interfaces.

[49] Graf C., van Blaaderen A. (2002). Metallodielectric Colloidal Core-Shell Particles for Photonic Applications. Langmuir.

[50] Schneider H.A. (2005). Polymer class specificity of the glass temperature. Polymer.

[51] Viel B., Ruhl T., Hellmann G.P. (2007). Reversible Deformation of Opal Elastomers. Chem. Mater..

[52] Pursiainen O.L.J., Baumberg J.J., Winkler H., Viel B., Spahn P., Ruhl T. (2007). Nanoparticle-tuned structural color from polymer opals. Opt. Express.

[53] Katritzky A.R., Sild S., Karelson M. (1998). Correlation and Prediction of the Refractive Indices of Polymers by QSPR. J. Chem. Inf. Comp. Sci..

[54] Yao W., Li Y., Huang X. (2014). Fluorinated poly(meth)acrylate: Synthesis and properties. Polymer.

[55] Gleinser W., Maier D., Schneider M., Weese J., Friedrich C., Honerkamp J. (1994). Estimation of sphere-size distributions in two-phase polymeric materials from transmission electron microscopy data. J. Appl. Polym. Sci..

[56] De Francisco R., Tiemblo P., Hoyos M., González-Arellano C., García N., Berglund L., Synytska A. (2014). Multipurpose Ultra and Superhydrophobic Surfaces Based on Oligodimethylsiloxane-Modified Nanosilica. ACS Appl. Mater. Interfaces.

[57] García-Domenech R., de Julián-Ortiz J.V. (2002). Prediction of Indices of Refraction and Glass Transition Temperatures of Linear Polymers by Using Graph Theoretical Indices. J. Phys. Chem. B.

[58] Schäfer C.G., Lederle C., Zentel K., Stuhn B., Gallei M. (2014). Utilizing stretch-tunable thermochromic elastomeric opal films as novel reversible switchable photonic materials. Macromol. Rapid Commun..

[59] Hart S.J., Terray A.V. (2003). Refractive-index-driven separation of colloidal polymer particles using optical chromatography. Appl. Phys. Lett..

[60] Aralaguppi M.I., Jadar C.V., Aminabhavi T.M. (1999). Density, Viscosity, Refractive Index, and Speed of Sound in Binary Mixtures of Acrylonitrile with Methanol, Ethanol, Propan-1-ol, Butan-1-ol, Pentan-1-ol, Hexan-1-ol, Heptan-1-ol, and Butan-2-ol. J. Chem. Eng. Data.

[61] Awwad A.M., Al-Dujaili A.H. (2001). Density, Refractive Index, Permittivity, and Related Properties for N-Formylmorpholine + Ethyl Acetate and + Butanone at 298.15 K. J. Chem. Eng. Data.

[62] Butt H.J., Jaschke M. (1995). Calculation of thermal noise in atomic force microscopy. Nanotechnology.

