# Supplementary material for: Fluoropolymer-Containing Opals and Inverse Opals by Melt-Shear Organization
Source: Molecules. 2019 Jan 17;24(2):333. doi: 10.3390/molecules24020333 (PMC6359200; doi:10.3390/molecules24020333)
Supplement: Supplementary file 1 [file molecules-24-00333-s001.pdf]

Supporting Information for

Fluoropolymer-Containing Opals and Inverse Opals by Melt-Shear Organization

Julia Kredel [1], Christian Dietz [2] and Markus Gallei [1,*]

[1] Ernst-Berl Institute of Technical and Macromolecular Chemistry, Technische Universität Darmstadt, Alarich-Weiss-Straße 4, 64287 Darmstadt, Germany;

[2] Institute of Materials Science, Physics of Surfaces, Technische Universität Darmstadt, Alarich-Weiss-Str. 2, D-64287 Darmstadt, Germany

Correspondence: m.gallei@mc.tu-darmstadt.de

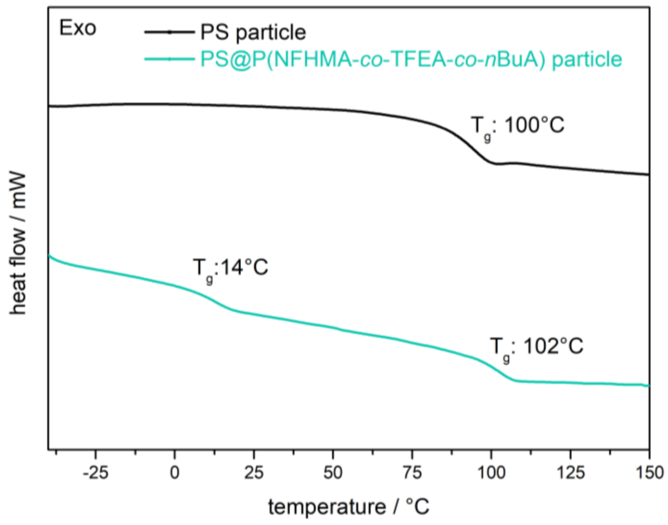

Figure S1: Differential Scanning Calorimetry (DSC) thermograms of PS@P(NFHMA-*co*-TFEA-*co*-*n*BuA) precipitated core/interlyer/shell particles (green) with two glass transition temperatures at 14 °C and 102 °C and of PS core particles (black) with the glass transition temperature of 100°C.

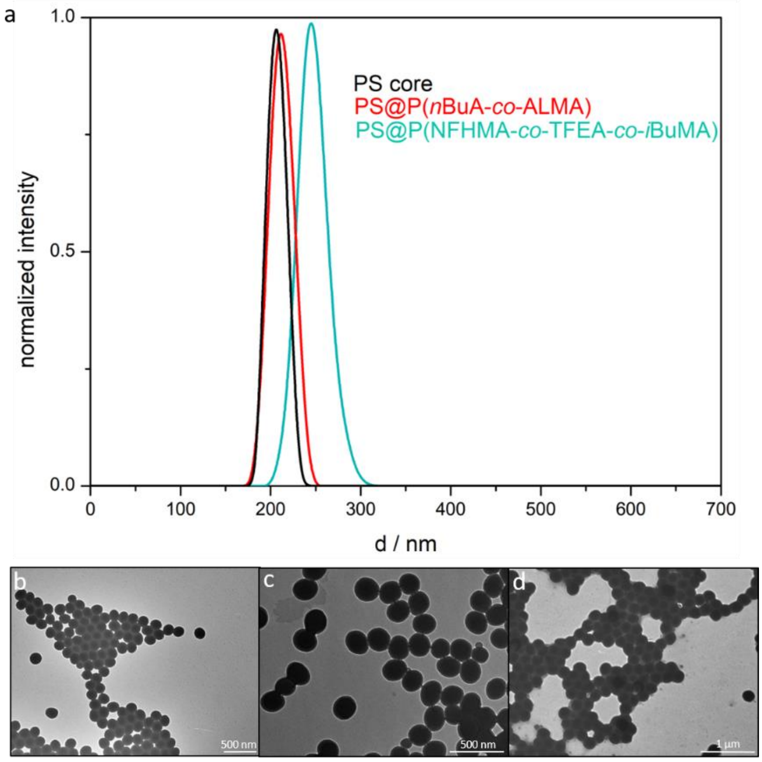

Figure S2: a) DLS investigations after each stage of stepwise emulsion polymerization to determine the hydrodynamic diameter and size distribution of the PS@P(NFHMA-*co*-TFEA-*co*-*n*BuA) particles; b) TEM images of PS cores; c) TEM image of the core/interlayer particles; d) TEM image of the core/interlayer/shell particles PS@P(NFHMA-*co*-TFEA-*co*-*n*BuA).

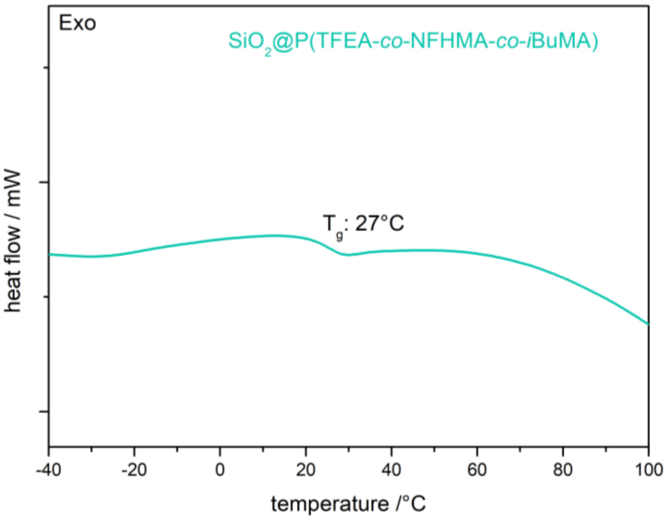

Figure S3: Differential Scanning Calorimetry (DSC) thermogram of SiO_2@P(TFEA-*co*-NFHMA-*co*-*i*BuMA) precipitated core/interlyer/shell particles with a glass transition temperature of 27° C.

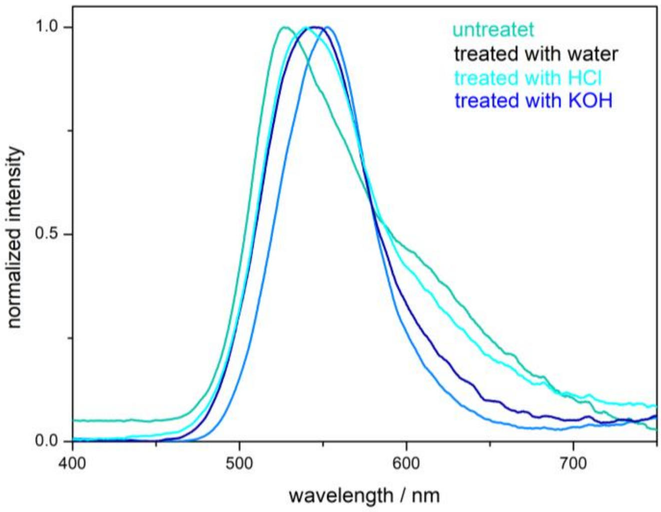

Figure S4: UV/-Vis spectra of the PS@P(NFHMA-*co*-TFEA-*co*-*n*BuA) opal film, untreated and treated with water, potassium hydroxide and hydrochloric acid. The reflection peak maxima untreated is located at a wavelength of 525 nm, treated with water at 544 nm, with potassium hydroxide (pH=13) 550 nm and hydrochloric acid (pH=1) at 540 nm.

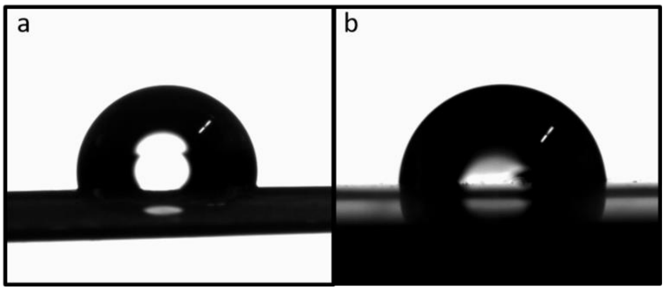

Figure S5: a) Photograph of a drop of water (2µl) on the PS@P(NFHMA-*co*-TFEA-*co*-*n*BuA) opal film with a contact angle of 106 °± 3°; b) photograph of a drop of water (2µl) on the SiO_2@P(NFHMA-*co*-TFEA-*co*-*n*BuA) inverse opal film with a contact angle of 102° ± 2°.

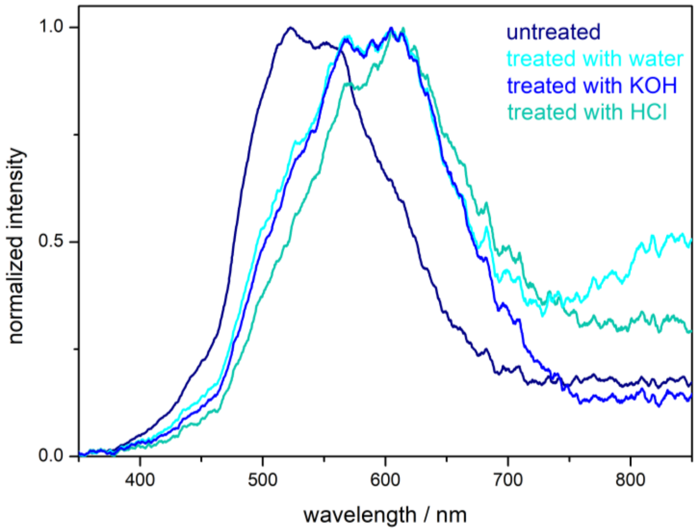

Figure S6: UV/-Vis spectra of the SiO_2@P(TFEA-*co*-NFHMA-*co*-*i*BuMA) opal film, untreated and treated with water, potassium hydroxide and hydrochloric acid. The reflection peak maxima untreated is located at a wavelength of 518 nm, treated with water at 570 nm, with potassium hydroxide (pH=13) 570 nm and hydrochloric acid (pH=1) at 574 nm.
